# Edentulous Patient Satisfaction with Conventional Complete Dentures

**DOI:** 10.3390/medicina58030344

**Published:** 2022-02-24

**Authors:** Una Soboleva, Irena Rogovska

**Affiliations:** 1 Department of Prosthodontics, Riga Stradinš University, LV-1007 Riga, Latvia; 2 Department of Doctoral Studies, Riga Stradinš University, LV-1007 Riga, Latvia; irena.rogovska@rsu.lv

**Keywords:** complete dentures, overall satisfaction, edentulous jaw, conventional dentures, denture aesthetics, bone resorption

## Abstract

*Background and Objectives*: Edentulism is considered to be an impediment impacting both patients’ quality of life and their nutrition. Conventional complete dentures are still a preferred treatment. However, there is no consensus on the most important factors which could substantially reduce the risk of patient dissatisfaction. This study evaluated the following determinants concerning patient satisfaction with complete maxillary and mandibular dentures: sex, denture-related functional and aesthetic aspects, and the degree of bone resorption. *Materials and Methods*: This study included 70 patients aged 34–81 years of age. All complete dentures were made by following the same technology. Visual analogue scales were used to assess patients’ overall satisfaction with dentures, comfort, ability to speak and chew, denture aesthetics, stability, and ease of prosthesis cleaning. Satisfaction with upper and lower dentures was rated separately. The degree of bone resorption was classified by using the Kalk and de Baat (1989) method. *Results*: The mean (SD) age of the study participants was 67.3 (10.4) years; 65.7% (*n* = 46) were females and 34.3% (*n* = 24) were males. There were no significant sex-based differences in resorption of the maxilla or mandibula. There were significant differences between maxillary and mandibular dentures, with lower mean satisfaction scores concerning chewing and maxillary complete dentures, and in regard to stability and comfort for mandibular complete dentures. There was a non-significant overall lower satisfaction with increased age. In multivariate analysis for mandibular complete dentures, aesthetics and stability significantly predicted the patient’s comfort levels, and the patient’s comfort significantly predicted overall satisfaction. For maxillary complete dentures, patient comfort and aesthetics significantly predicted overall patient satisfaction. *Conclusions*: Age, sex, and degree of resorption were not associated with patient satisfaction with complete dentures. Overall, patient satisfaction with both maxillary and mandibular complete dentures was related to their comfort level and denture aesthetics, and patient comfort itself was associated with stability of the mandibular denture.

## 1. Introduction

Despite a declining prevalence of edentulism, the proportions of edentulous patients will not likely decrease due to ageing of societies [1]. Edentulism is considered an impediment impacting both patients’ quality of life and their nutrition. Globally, substantial proportions of edentulous individuals, particularly among the elderly, are in need of rehabilitation. Most studies found that mandibular implant overdentures were superior to complete dentures regarding patient satisfaction and quality of life [2]. In spite of the fact patients were offered free implants for mandibular overdentures, 36% of them refused, with the most common reasons being a fear of surgical risks and a belief that implants were unnecessary when complete dentures functioned well [3].

The demographic data on population ageing show that the need to rehabilitate edentulous patients will remain considerable for many more decades. Conventional complete dentures are still a preferred treatment for edentulous patients, and this treatment modality improves oral-health-related quality of life [4,5]. Complete dentures are commonly accepted, as they provide an expected aesthetic and enable patients to maintain normal speech, as well as provide occlusal support for adequate chewing. Such dentures should be comfortable and lead to patient satisfaction [1], both of which are considered the main goals of treatment. In previous studies, satisfaction with complete dentures was associated with several factors. However, there is still no consensus on the most important factors, which could substantially reduce the risk of patient dissatisfaction. In general, more focus has been given to mandibular dentures, which are traditionally considered to be more problematic.

It is important to identify what specifically determines patient satisfaction with either maxillary or mandibular dentures and to assess whether these determinants differ between the two jaws. Such identification may enhance patient satisfaction and denture acceptance, consequently improving patient well-being and quality of life.

This study evaluated the following determinants concerning patient satisfaction with complete maxillary and mandibular dentures: sex, denture-related functional and aesthetic aspects, and the degree of bone resorption.

## 2. Materials and Methods

During a one-year period, the current cohort study recruited 70 edentulous patients (46 females and 24 males) aged 34–81 years, for whom conventional complete dentures for both jaws were made at the Prosthodontic Clinic at the Institute of Stomatology, Riga Stradins University (RSU). The study was approved by the Riga Stradins University Research Ethics Committee: Approval Code, 2-PĒK-4/49/2022; Approval Date, 08 February 2022.

The standardization of the study conditions included the following: all complete dentures were made at the same dental laboratory, following the standardized study protocol, including taking primary and secondary impressions; recording jaw relationships, using occlusal wax rims; and setting artificial teeth in a mutually balanced occlusion scheme, using one or two trials for insertion and fitting of the dentures. Adjustments to the dentures were made during the follow-up appointment. Two months after denture use, all patients were invited to participate in a follow-up survey about their satisfaction with the dentures. All 70 patients came for the visit.

Different aspects of patient satisfaction were assessed separately for either upper and lower dentures employing the 100 mm Visual Analogue Scales (VAS). The following aspects of patient satisfaction were considered: patient level of comfort; ability to chew and speak; and denture stability, aesthetics, and ease of cleaning. Patients’ responses were collected without the supervision/guidance from a dentist. The following questions (Q) (range of responses) were asked: (Q1) Are you satisfied with your dentures? (‘very dissatisfied’ to ‘very satisfied’); (Q2) Do you feel comfortable using dentures? (‘absolutely no’ to ‘perfectly comfortable’); (Q3) Are you happy with the way you look with your dentures? (‘very dissatisfied’ to ‘very satisfied’); (Q4) Can you chew the food? (‘very badly’ to ‘very good’); (Q5) Do your dentures cause any trouble when speaking? (‘very large disturbances’ to ‘absolutely none’); (Q6) Are your dentures stable? (‘very unstable’ to ‘very stable’); (Q7) Is everyday care of your dentures easy to provide? (‘very difficult’ to ‘very easy’).

The degree of bone resorption was based on the evaluation of the anatomical stone casts of a patient’s upper and lower jaws, using the classification system described by Kalk and de Baat (1989). This evaluation was performed by a well-experienced specialist.

For the mandible, the following criteria were used:

Class 0: Moderate resorption; both the genial tubercle and the mylohyoid lines are below the level of the alveolar ridge.

Class 1: High degree of resorption; the genial tubercle and the mylohyoid lines are either just below the highest point of the alveolar ridge or at the same level.

Class 2: Extensive resorption; the genial tubercle is above the level of the alveolar ridge, and the mylohyoid lines are at the same level or above the alveolar ridge.

The following criteria were used for the maxilla:

Class 0: Little, if any, resorption, with there being a difference in height between the lowest point on the mucosal membrane and the highest point on the alveolar ridge; both left and right sides show well-developed maxillary tuberosities and there is a clear difference in height between the lowest point palatally and the highest point on the alveolar ridge. There is no “flabby ridge”.

Class 1: Extensive degree of resorption; the alveolar ridge is narrow and there is little difference in height between the lowest points on the mucosal membrane and the palate and the highest point on the alveolar ridge. The palate is low and the maxillary tuberosities are only moderate in size. There may be a flabby ridge [6].

### Statistical Analysis

The data were analyzed by using descriptive and analytical statistical methods. Mean values and standard deviations were calculated for all aspects of patient satisfaction. Differences between groups were tested by using the Mann–Whitney test, Chi-Square test, and Wilcoxon test. Overall patient satisfaction was used as the dependent variable, while level of comfort, chewing, stability, aesthetics, speaking ability, and cleaning of dentures were the independent variables. Age, sex, and bone-resorption level were also tested as potential determinants of overall patient satisfaction. Univariate regression analyses evaluated the individual effect of each independent variable on both overall and comfort satisfaction. In addition, multivariate regression analyses estimated the combined effect of several determinants of patient satisfaction and patient comfort. Statistical significance was set at *p* < 0.05.

## 3. Results

The mean age of the study participants was 67.3 (± 10.4) years; 65.7% (*n* = 46) were females and 34.3% (*n* = 24) were males. Although the mean age of females (68.4 ± 1.4) was higher than that of males (65.3 ± 2.5), this difference was not statistically significant (*p* = 0.241). There were no male patients in the 50–55-year age group, while the patient distributions in other age groups were similarly balanced between the two sexes. In maxilla, 57% (*n* = 40) of patients had a Class 0 resorption level, and 30% (*n* = 30) had a Class 1 resorption level. For mandibula, 18.6% (*n* = 13) of patients had a Class 0 resorption level, while 47.1% (*n* = 33) had a Class 1 resorption level and 34.3% (*n* = 24) had a Class 2 resorption level. There were no statistically significant sex-based differences in either maxilla or mandibula resorption levels (Table 1).

Patient satisfaction with maxillary and mandibular complete dentures for both sexes is presented in Table 2.

There were no statistically significant sex differences in any of the specific satisfaction aspects with either maxillary or mandibular complete dentures.

There were significant differences in satisfaction (Table 3), where lower satisfaction was indicated for chewing with maxillary complete dentures and for stability and comfort with mandibular complete dentures. Although overall satisfaction, as well as satisfaction with chewing/mastication, speaking, and stability while wearing maxillary and mandibular complete dentures, decreased with age, this trend was not statistically significant.

Univariate regression analysis examined individual effects of different factors in relationship to overall satisfaction with complete mandibular and maxillary dentures. There were no significant effects of age (F (1, 68) = 0.14, *p* = 0.706), sex (F (1, 68) = 0.44, *p* = 0.511), or level of resorption on overall satisfaction with complete mandibular dentures. Although overall satisfaction decreased with age, this trend was not statistically significant.

Similarly, there were no significant effects of age (F (1, 68) = 0.02, *p* = 0.891), sex (F (1, 68) = 1.28, *p* = 0.26), or level of resorption on overall satisfaction with complete maxillary dentures.

A multiple regression analysis was run to predict patient’s overall satisfaction with complete mandibular and maxillary dentures (see Table 4). The only variable that statistically significantly predicted overall patient satisfaction with mandibular dentures was patient comfort (F (6, 63) = 2074, *p* < 0.001, *R^2^* = 0.664), while for maxillary complete dentures, it was patient comfort and aesthetics (F (6, 63) = 32.84, *p* < 0.001, *R*^2^ = 0.735).

Univariate regression analysis examined the effect of different predictors on patient comfort with complete mandibular dentures. There were no significant effects of age (F (1, 68) = 0.92, *p* = 0.340), sex (F (1, 68) = 0.26, *p* = 0.615), or bone resorption level on patient comfort, while patient comfort significantly related to aesthetics, patient’s chewing and speaking ability, denture stability, and cleaning.

There were no significant effects of sex (F (1, 68) = 0.19, *p* = 0.661), age (F (1, 68) = 0.02, *p* = 0.895), or level of resorption on patient comfort, while patient comfort was significantly related to chewing, speaking, denture stability, and cleaning.

A multiple regression analysis was run to predict patient comfort with complete mandibular and maxillary dentures (see Table 5). Aesthetics and stability were significant predictors of patient comfort with complete mandibular dentures (F (6, 64) = 22.34, *p* < 0.001, *R*^2^ = 0.607), while only aesthetics was a statistically significant predictor of patient comfort with complete maxillary dentures (F (5, 64) = 21.01, *p* < 0.001, *R^2^* = 0.591).

## 4. Discussion

Treatment with complete dentures is still the first choice for tooth replacement in edentulous individuals [7,8]. Patient satisfaction with complete dentures is important for a patient’s overall quality of life. Therefore, it is important to identify factors related to satisfaction. It has been recommended that researchers conduct more studies that use patient satisfaction as the primary outcome measure in treatment evaluation and that more attention needs to be paid to understand which aspects of patient satisfaction indicate successful treatment outcomes [9]. Several previous studies mainly examined the objective parameters of denture quality and functionality; however, professional viewpoints often do not correspond with patients’ perceptions. Therefore, our research focused on patient perspectives, and patient self-evaluation of their dentures was measured by asking specific questions, the responses to which were rated on VAS from worst to best.

Our sample was relatively small, as it was confined by the following inclusion criteria: patients having both edentulous jaws, consenting to participate, and choosing conventional complete dentures for both jaws. In preparation for the study, we pre-screened a large number of patients; however, the majority of these patients had only one edentulous jaw or preferred implant-supported overdentures. Moreover, to ensure the homogeneity of the study population, we excluded patients with previously made and long-term use of conventional dentures. This way, during the period of a year, the current cohort study recruited 70 edentulous patients.

In previous studies, the most common follow-up period was between 2 and 3 months after the delivery of complete dentures [10,11,12], as this length of time gives patients sufficient time to adapt to their prostheses. One possible limitation of the current study is that it did not distinguish between whether patients had complete dentures for the first time or if they already had previous denture-related experience. Regardless, our study patients had either experience with partial removable dentures or previous experience with worn-out complete dentures; therefore, most likely, previous denture-related experience did not have a substantial influence on patient satisfaction with complete dentures.

The importance of occlusion has been discussed in several publications. Significantly less patient satisfaction in terms of comfort, stability, and retention of complete dentures with partially group-function occlusion was reported as compared to complete dentures with balanced occlusal schemes, such as buccalised occlusion [13,14]. Similarly, Abduo (2013) systematic review and Poštić et al. (1992) study concluded that bilaterally balanced occlusion or lingualized bilaterally balanced occlusion was equally acceptable to patients in relation to their masticatory ability, esthetics, comfort, and speech. There is some evidence that lingualized bilaterally balanced occlusion is beneficial for patients with severely resorbed ridges in terms of mastication and denture stability [15,16]. However, Goldstein et al.’s (2021) review identified contradictory evidence, Poštić et al. (1992) and another study reported patients satisfied with balanced occlusal schemes [16,17], and other studies did not associate satisfaction differently with either balanced or non-balanced occlusion of complete dentures [17]. Despite that some studies reported that the quality of the dentures, occlusion, tooth positioning, and prosthesis manufacturing protocol did not influence masticatory efficiency [18] or patient satisfaction with complete dentures [13,19,20,21,22,23], we decided to use the standardized protocol for manufacturing all dentures in one dental laboratory, aiming to exclude any potential influences or variations in the prosthesis manufacturing techniques. The same approach was applied to all dentures, including the setting of teeth in the same occlusion scheme, namely a mutually balanced occlusion. Furthermore, previous studies indicated that the presence or establishment of a good relationship between a dentist and patient, along with adequate counselling before the initiation of the treatment, is more important for patient satisfaction than providing dentures with all the sophisticated technical specifications [7,20,24]. The increased focus on patient well-being during the study and personalized invitations for follow-up visits could make patients feel more cared for, possibly providing higher levels of patient satisfaction.

Overall, patients were less satisfied with mandibular dentures compared with maxillary dentures in almost all parameters, except for speaking and chewing, where the maxillary dentures were evaluated less positively by female patients. Patients had the least problems with cleaning their maxillary dentures, and this aspect of satisfaction received the highest overall rating. One unexpected finding was that chewing was the most difficult issue associated with maxillary dentures. The mandibular dentures did not interfere with speech and were easy to clean, but their lack of stability caused problems, which hindered patient comfort. This finding can be explained by the anatomical differences between the maxilla and mandible, and increased difficulties with denture retention in the lower jaw. Influence of the upper frontal (incisor) area on phonetics possibly interfered with speech, due to maxillary dentures. Unfortunately, there have been no previous studies comparing dentures between the two jaws, as most of the previous studies focused on mandibular dentures. Therefore, further studies are necessary to validate our results.

In this study, we also aimed to examine if there were any sex-based differences. There were no significant sex-related differences, although women were slightly less satisfied. Women were less satisfied than men with aesthetics; this finding is in line with results of previous studies [23,25]. Females were also less satisfied than men with the chewing ability of upper and lower dentures, as well as with their comfort. Previous studies showed that men adapt more rapidly to new dentures than women [26], but they have more objections regarding mastication [23]. Our findings suggest the opposite trend, where men were more satisfied with chewing ability than women. This could be explained by differences in mean ages between the two sexes; the mean age of males was lower than that of females. Moreover, there were no males in the 50–55 age group. This sex-related difference might be explained by the higher proportion of women in our sample and possibly females being more concerned than males about oral-health-related quality of life [11,27].

Although all consecutive consenting eligible patients were included in the study, the similar sex-based proportions were not achieved. We assume that this disproportionate sex-based distribution in our study sample was due to the following reasons: dentures are needed for older adults, and, in Latvia, there were proportionally more older women than men, and women were less likely to choose surgery and implants, or they tended to change their dentures more frequently; thus, the cost of dental treatments for women was an important consideration. Given that our sample included all eligible consecutive patients, we think our study findings are applicable to other similar patients. However, the generalization of our findings should be interpreted with caution, because we did not control for education and occupation, which might have had an influence on patient comfort and satisfaction.

Previous studies have attempted to connect ridge form to prosthodontic success. It is believed that the lower the alveolar bone resorption level, the better the retention and stability of the prosthesis, and consequently, the better function of the prostheses and improved patient satisfaction. To characterize levels of bone resorption, we used a classification system developed to easily determine the level of resorption in clinical practice. Despite the fact that women in the study corresponded to the age group of increased risk for osteoporosis, which has also been associated with increased bone loss in the jaw bones, we did not observe any sex differences in level of bone resorption [28,29]. Moreover, we did not find a direct association between decreased patient satisfaction and higher levels of bone resorption. Patient satisfaction was lower for the stability of the mandibular prosthesis and for chewing with the maxillary prosthesis, and this would clinically suggest that there may be an association with bone resorption, but this was not confirmed by our findings. This is also in accordance with other studies which did find such an association [19,30,31]. No strong correlation has been shown to exist between the quality of the denture-supporting tissues and the outcome of complete denture treatment and patient satisfaction level. Da Conceição Araújo et al. (2018) evaluated the effect of objectively assessed denture quality on user satisfaction with complete dentures during a 5-year follow-up period, and researchers concluded that anatomical characteristics of the alveolar ridge were not associated with complete denture use, while patient satisfaction was the most important factor for patient well-being [32].

Several studies examined the effect of age as a possible determinant of prosthodontic success; it is known that elderly patients have decreased coordination of the oral muscles, as well as reduced resistance to pressure in the mucosal tissues [33,34]. A single study reported that patients over 60 years of age had more difficulties in adapting to a new set of dentures than their younger counterparts [35]. Similarly, a significant difference in tongue motor function was found between those younger than 80 and those 80 years or older [36]. In our study, there was a trend toward a decrease in function satisfaction with increasing age, but this was not statistically significant. Although we included a wide age range of patients, a majority of them were 60 to 69 years of age. Many of the problems of aging may not yet have manifested in this population. Our results are in line with the available evidence that age cannot be considered a positive or negative prognostic indicator for a successful outcome [7,20,37].

If associations between the supposed anatomical and technical prerequisites for successful treatment and patient satisfaction with complete dentures are weak or lacking and none of these parameters had a conclusive effect on patient assessment, then a patient’s subjective opinion may be the most important factor for the assessment of treatment result [32].

We chose to examine determinants of patient satisfaction tested in previous studies, such as comfort with dentures, their aesthetics, ability to chew and speak, denture stability, and ease of cleaning. Looking at both overall satisfaction and separate specific satisfaction aspects, their importance cannot be denied. However, when controlling for other factors, only patient comfort was associated with overall patient satisfaction involving both mandibular and maxillary complete dentures. A similar finding was reported by Awad and Feine (1998). The highest proportion of patients reported comfort as the most important quality aspect of mandibular complete dentures, followed by the ability to chew and denture stability [9]. Our findings indicate that comfort with mandibular dentures was significantly associated not only with their stability, but also, as in the upper jaw, their aesthetics. This is in agreement with other studies where loss of retention and stability with mandibular complete dentures usually caused discomfort and functional limitations [11], along with an impact on masticatory ability, as indicated by objective and subjective assessments [38]. Although the aesthetics of dentures were slightly more important for women, this was significantly associated with patient comfort. Our findings are in line with Awad and Feine (1998), who found that patient satisfaction with conventional complete dentures was dependent on the appearance and functionality of the appliance [9].

In addition, a patient’s socioeconomic status, previous denture experience, and some personality characteristics might, at least in part, determine patient satisfaction; however, these factors were not analyzed in our study. Instead, we were interested in general trends that could be applied to any potential patient in any practice. To obtain a representative study population, it was important that both jaws were edentulous in the patients, and that complete dentures for both jaws were made simultaneously. Unfortunately, these inclusion criteria limited the sample size, and this was an important limitation of our study.

Overall, our study findings support the previous findings that a majority of complete denture wearers are satisfied with the benefits provided by their dentures.

## 5. Conclusions

1. Overall satisfaction was related to a patient’s comfort levels when using both maxillary and mandibular complete dentures.
2. The aesthetics of maxillary and mandibular dentures played an important role in ensuring patient comfort.
3. The stability of the mandibular dentures was associated with patient comfort.
4. Sex differences concerning satisfaction with complete dentures were not observed.
5. Increased age and bone resorptions level had no influence on satisfaction levels.

## Figures and Tables

**Table 1:** Maxillary and mandibular bone-resorption levels by sex.

Bone Resorption	Females (*n* = 46)	Males (*n* =2 4)	*p*-Values ^#^
	N (%)	N (%)	
Maxilla			
Class 0	27 (67.5)	13 (32.5)	
Class 1	19 (63.3)	11 (36.7)	0.800
Mandibula			
Class 0	9 (69.2)	4 (30.8)	
Class 1	22 (66.7)	11 (33.3)	
Class 2	15 (62.5)	9 (37.5)	0.890

^#^ Chi-Square test.

**Table 2:** Satisfaction with maxillary and mandibular complete dentures by sex.

Aspects of Satisfaction	Females (*n* = 46)	Males (*n* = 24)	*p*-Values ^#^
	Mean (SD)	Mean (SD)	
Maxillary dentures			
Overall satisfaction	8.2 (1.99)	8.7 (1.38)	0.301
Comfort	8.4 (1.76)	8.6 (1.46)	0.385
Aesthetics	8.4 (1.91)	8.7 (1.82)	0.197
Chewing/mastication	6.2 (2.74)	6.9 (2.06)	0.560
Speaking	8.1 (2.34)	8.2 (2.02)	0.896
Stability	8.6 (1.53)	8.1 (1.66)	0.281
Cleaning	8.5 (1.39)	8.6 (0.89)	0.682
Mandibular dentures			
Overall satisfaction	6.8 (2.81)	7.3 (2.56)	0.461
Comfort	5.9 (2.95)	6.3 (2.38)	0.877
Aesthetics	8.1 (2.35)	8.1 (2.64)	0.406
Chewing/mastication	8.1 (2.07)	8.6 (1.33)	0.270
Speaking	8.7 (1.36)	8.8 (0.95)	0.980
Stability	5.7 (2.89)	5.8 (2.56)	0.970
Cleaning	8.4 (1.75)	8.4 (1.03)	0.289

^#^ Mann–Whitney test.

**Table 3:** Different aspects of patient satisfaction with maxillary and mandibular complete dentures.

Aspects of Satisfaction	Maxillary Dentures	Mandibular Dentures	*p*-Values ^#^
	Mean (SD)	Mean (SD)	
Overall satisfaction	8.4 (1.81)	7.0 (2.72)	<0.001
Comfort	8.5 (1.76)	6.0 (2.87)	<0.001
Aesthetics	8.5 (1.87)	8.1 (2.43)	0.011
Chewing/mastication	6.5 (2.52)	8.2 (1.86)	<0.001
Speaking	8.1 (2.22)	8.7 (0.14)	<0.001
Stability	8.4 (1.65)	5.7 (2.77)	<0.001
Cleaning	8.6 (1.23)	8.4 (1.53)	0.028

^#^ Wilcoxon test.

**Table 4:** Multivariate regression analysis of overall satisfaction with mandibular and maxillary complete dentures.

Predictors	Standardized Coefficients	95% CI	*p*-Values
Mandibular dentures			
Comfort	0.546	0.308–0.784	<0.001
Maxillary dentures			
Comfort	0.770	0.550–0.990	<0.001
Aesthetics	0.247	0.070–0.424	0.007

**Table 5:** Multivariate regression analysis of comfort with mandibular and maxillary complete dentures.

Predictors	Standardized Coefficient	95% CI	*p*-Values
Mandibular dentures			
Aesthetics	0.253	0.035–0.471	0.024
Stability	0.648	0.478–0.818	<0.001
Maxillary dentures			
Aesthetics	0.398	0.224–0.572	<0.001

## Data Availability

The data presented in this study are available on request from the corresponding author. The data are not publicly available due to privacy restrictions.

## References

[1] Zou Y., Zhan D. (2015). Patients’ expectation and satisfaction with complete denture before and after the therapy. Vojn. Pregl..

[2] Assunção W.G., Barão V.A., Delben J.A., Gomes E.A., Tabata L.F. (2010). A comparison of patient satisfaction between treatment with conventional complete dentures and overdentures in the elderly: A literature review. Gerodontology.

[3] Walton J.N., MacEntee M.I. (2005). Choosing or refusing oral implants: A prospective study of edentulous volunteers for a clinical trial. Int. J. Prosthodont..

[4] Adam R.Z., Geerts G.A., Lalloo R. (2007). The impact of new complete dentures on oral health-related quality of life. S. Afr. Dent. J..

[5] Ellis J.S., Pelekis N.D., Thomason J.M. (2007). Conventional rehabilitation of edentulous patients: The impact on oral health-related quality of life and patient satisfaction. J. Prosthodont..

[6] Kalk W., de Baat C. (1989). Some factors connected with alveolar bone resorption. J. Dent..

[7] McCunniff M., Liu W., Dawson D., Marchini L. (2017). Patients’ esthetic expectations and satisfaction with complete dentures. J. Prosthet. Dent..

[8] Thomason J.M., Kelly S.A., Bendkowski A., Ellis J.S. (2012). Two implant retained overdentures--a review of the literature supporting the McGill and York consensus statements. J. Dent..

[9] Awad M.A., Feine J.S. (1998). Measuring patient satisfaction with mandibular prostheses. Community Dent. Oral Epidemiol..

[10] Awad M.A., Lund J.P., Shapiro S.H., Locker D., Klemetti E., Chehade A., Savard A., Feine J.S. (2003). Oral health status and treatment satisfaction with mandibular implant overdentures and conventional dentures: A randomized clinical trial in a senior population. Int. J. Prosthodont..

[11] Cardoso R.G., De Melo L.A., Barbosa G.A.S., Calderon P.D.S., Germano A.R., Junior W.M., Carreiro A. (2016). Impact of mandibular conventional denture and overdenture on quality of life and masticatory efficiency. Braz. Oral Res..

[12] Seenivasan M.K., Banu F., Inbarajan A., Natarajan P., Natarajan S., Anand Kumar V., Seenivasan M. (2019). The Effect of Complete Dentures on the Quality of Life of Edentulous Patients in the South Indian Population Based on Gender and Systemic Disease. Cureus.

[13] Moradpoor H., Salari F., Ebadian B., Raissi S., Shirani M. (2018). Patient satisfaction with occlusal scheme of conventional complete dentures: A randomised clinical trial (Part II). J. Oral Rehabil..

[14] Moradpoor H., Salari F., Mosharraf R., Raissi S., Shirani M. (2020). Patient satisfaction with occlusal scheme of conventional complete dentures. J. Oral Rehabil..

[15] Bduo J. (2013). Occlusal Schemes for Complete Dentures: A Systematic Review. Int. J. Prosthodont..

[16] Poštić S., Krstić M., Teodosijević M. (1992). A comparative study of the chewing cycles of dentate and denture-wearing subjects. Int. J. Prosthodont..

[17] Goldstein G., Kapadia Y., Campbell S. (2021). Complete denture occlusion: Best evidence consensus statement. J. Prosthodont..

[18] Ribeiro J.A.M., Machado de Resende C.M.B., Lopes A.L.C., Mestriner W., Roncalli A.G., Farias-Neto A., da Fonte Porto Carreiro A. (2012). Evaluation of Complete Denture Quality and Masticatory Efficiency in Denture Wearers. Int. J. Prosthodont..

[19] Carlsson G.E., Omar R. (2010). The future of complete dentures in oral rehabilitation. A critical review. J. Oral Rehabil..

[20] Marchini L. (2014). Patients’ satisfaction with complete dentures: An update. Braz. Dent. Sci..

[21] Regis R.R., Cunha T.R., Della Vecchia M.P., Ribeiro A.B., Silva-Lovato C.H., de Souza R.F. (2013). A randomised trial of a simplified method for complete denture fabrication: Patient perception and quality. J. Oral Rehabil..

[22] Kawai Y., Muarakami H., Feine J.S. (2018). Do traditional techniques produce better conventional complete dentures than simplified techniques? A 10-year follow-up of a randomized clinical trial. J. Dent..

[23] Mysore A.R., Aras M.A. (2012). Understanding the psychology of geriatric edentulous patients. Gerodontology.

[24] Komagamine Y., Kanazawa M., Sasaki Y., Sato Y., Minakuchi S. (2017). Prognoses of new complete dentures from the patient’s denture assessment of existing dentures. Clin. Oral Investig..

[25] Al-Omiri M.K., Sghaireen M.G., Al-Qudah A.A., Abu Hammad O., Lynch C.D., Lynch E. (2014). Relationship between impacts of removable prosthodontic rehabilitation on daily living, satisfaction and personality profiles. J. Dent..

[26] Panek H., Krawczykowska H., Dobosz A., Napadłek P., Panek B.A., Sosna-Gramza M. (2006). Follow-up visits as a measure of adaptation process to removable prostheses. Gerodontology.

[27] Sivakumar I., Sajjan S., Ramaraju A.V., Rao B. (2015). Changes in Oral Health-Related Quality of Life in Elderly Edentulous Patients after Complete Denture Therapy and Possible Role of their Initial Expectation: A Follow-Up Study. J. Prosthodont..

[28] Ozola B., Slaidina A., Laurina L., Soboleva U., Lejnieks A. (2011). The influence of bone mineral density and body mass index on resorption of edentulous jaws. Stomatologija.

[29] Springe B., Slaidina A., Soboleva U., Lejnieks A. (2014). Bone mineral density and mandibular residual ridge resorption. Int. J. Prosthodont..

[30] Heydecke G., Klemetti E., Awad M.A., Lund J.P., Feine J.S. (2003). Relationship between prosthodontic evaluation and patient ratings of mandibular conventional and implant prostheses. Int. J. Prosthodont..

[31] Van Waas M.A. (1990). Determinants of dissatisfaction with dentures: A multiple regression analysis. J. Prosthet. Dent..

[32] Da Conceição Araújo M.M., Martins M.R., Dos Santos Soares A.R., de Abreu Carvalho L.R., Gomes V.E., Ferreira E.F., Miranda Cota L.O., Senna M.I.B., Ferreira R.C. (2018). Relationship Between Quality of Complete Dentures and User Satisfaction at 1 and 5 Years Postinsertion. Int. J. Prosthodont..

[33] Michael C.G., Javid N.S., Colaizzi F.A., Gibbs C.H. (1990). Biting strength and chewing forces in complete denture wearers. J. Prosthet. Dent..

[34] Komagamine Y., Kanazawa M., Yamada A., Minakuchi S. (2019). Association between tongue and lip motor functions and mixing ability in complete denture wearers. Aging Clin. Exp. Res..

[35] Diehl R.L., Foerster U., Sposetti V.J., Dolan T.A. (1996). Factors associated with successful denture therapy. J. Prosthodont..

[36] Kikutani T., Tamura F., Nishiwaki K., Kodama M., Suda M., Fukui T., Takahashi N., Yoshida M., Akagawa Y., Kimura M. (2009). Oral motor function and masticatory performance in the community-dwelling elderly. Odontology.

[37] Critchlow S.B., Ellis J.S. (2010). Prognostic indicators for conventional complete denture therapy: A review of the literature. J. Dent..

[38] Limpuangthip N., Somkotra T., Arksornnukit M. (2021). Subjective and objective measures for evaluating masticatory ability and associating factors of complete denture wearers: A clinical study. J. Prosthet. Dent..

